# Pattern of initial clinical manifestations of systemic lupus erythematosus in a tertiary care hospital

**DOI:** 10.12669/pjms.325.11480

**Published:** 2016

**Authors:** Shabnam Batool, Nighat Mir Ahmad, Muhammad Ahmed Saeed, Sumaira Farman

**Affiliations:** 1 Dr. Shabnam Batool, FCPS Medicine. Department of Rheumatology, Fatima Memorial Hospital Lahore, Pakistan; 2 Prof. Nighat Mir Ahmad, MD, FACP, FACR. Department of Rheumatology, Fatima Memorial Hospital Lahore, Pakistan; 3 Dr. Muhammad Ahmed Saeed, FCPS Medicine, FCPS Rheumatology. Department of Rheumatology, Fatima Memorial Hospital Lahore, Pakistan; 4 Dr. Sumaira Farman, MBBS, FRCP, FACP, FACR. Department of Rheumatology, Fatima Memorial Hospital Lahore, Pakistan

**Keywords:** Clinical manifestations, Pattern, Systemic Lupus Erythematosus

## Abstract

**Objective:**

To determine the pattern of initial clinical manifestations of Systemic Lupus Erythematosus (SLE) and to compare these features with those recorded elsewhere in Pakistan

**Methods:**

This cross-sectional, descriptive study was performed in the Department of Rheumatology, Fatima Memorial Hospital, Lahore, Pakistan, from November 2015 to January 2016. Sixty one patients of SLE diagnosed as per ACR (American College of Rheumatology) 1982 revised criteria, were enrolled. The patients were evaluated for the initial clinical manifestations of SLE. The information was collected on a specially designed proforma and analyzed by using SPSS version 17.

**Results:**

Out of 61 patients, 49 (80.3%) were females and 12 (19.7%) males, showing a female to male ratio of 4:1. The mean age of patients was 26.2 ± 7.9 years. Fatigue was the most common presenting feature in 56 (91.8%) patients, followed by joint pains in 55 (90.2%) and fever in 54 (88.5%). Renal involvement was found in 46 (75.4%). Comparison of these presenting features was made with other studies carried out in Northern Pakistan (Islamabad) and in central Punjab (Pakistan). There were statistically significant differences in fever, fatigue and arthritis between our patients and the other two above mentioned study groups. However, comparison of renal manifestations showed significant difference only with Islamabad study, and not with previous study from central Punjab.

**Conclusion:**

In this study, majority of patients presented with combination of fatigue, fever, rash and arthritis. Almost three-fourth of patients had renal manifestations at initial presentation. Therefore, it is important for clinicians to have high index of suspicion for SLE, when patients present with above symptoms.

## INTRODUCTION

Systemic Lupus Erythematosus (SLE) is the prototypic multisystem autoimmune disorder with a broad spectrum of clinical presentations encompassing almost all organs and tissues.^1^ Its presentation and course vary greatly ranging from indolent to fulminant.^2^ The majority of pathology in SLE is related to deposits of immune complexes in various organs, which trigger complement and other mediators of inflammation.^3^ Estimated incidence rates in U.S.A and Europe range 1-23 per 100,000 per year. Prevalence in adults is as high as 150 per 100,000 in United States and is 20-50 per 100,000 in Europe.^4^ Women are affected upto nine times more frequently than men.^5^ Sixty five percent of SLE patients have disease onset between the ages of 16 – 55, 20% present before 16 years, and 15% after the age of 55 years.^6^ There is a varying epidemiological information regarding SLE among Asian countries. Prevalence rates usually range within 30-50 per 100,000 population. Incidence rates, vary from 0.9 per 100,000 to 3.1% per annum.^7^

A comprehensive review of the available SLE epidemiologic data in Pakistan has not been performed so far.^8^ SLE is a complicated disease, as no patient presents with the same set of symptoms.^9^ This can be due to the surrounding environment, such as the climate, where sunlight plays a role in photosensitivity and skin rashes, or a colder climate can initiate complications such as Raynaud’s phenomenon.^9^ Symptoms vary widely as SLE is protean in its manifestations and follows a relapsing and remitting course.^9^,^10^ There are diverse abnormalities of skin, kidney, haematological, musculoskeletal, pulmonary, cardiovascular and neurological systems.^10^

So far, three studies have been conducted in Pakistan on clinical features of SLE at initial presentations, one study was conducted in Islamabad, and other two depicting initial clinical manifestatons in patients belonging to Karachi and central Punjab.^11^-^13^ This study aimed to find out spectrum of presenting features of SLE in our population and its comparison with studies conducted in Northern parts of Pakistan (Islamabad) and in the central Punjab.

## METHODS

This cross sectional study was performed in the Rheumatology Department of Fatima Memorial Hospital over a period of three months from November 2015 to January 2016. Total of sixty one patients of Systemic Lupus Erythematosus of 16 years of age or more, of both genders, who were diagnosed according to American College of Rheumatology (ACR) 1982 revised criteria, were included by purposive sampling technique. Patients not willing to give written consent or those having malignancies or any other chronic diseases were excluded. Signed informed consent was obtained from the patients and confidentiality was ensured. Prior approval by Institutional Review Board of the hospital was taken.

All patients were evaluated for the initial clinical manifestations of SLE. The information was collected on a specially designed proforma and analyzed by using SPSS version 17. The social demographic data was analysed and presented as frequency tables and diagrams. Means ± SD was determined for numerical variables, whereas, frequencies and percentages were computed for categorical variables. Since this was a descriptive study, no test of significance was required. However, Chi square test was applied to determine any significance amongst within the subjects of our findings, and between the earlier two studies carried out in Pakistan. A p-value of 0.05 or less was taken as statistically significant.

## RESULTS

Out of 61 patients, 49 (80.3%) were females and 12 (19.7%) males, showing a female to male ratio of 4:1. Among these patients, 21 (34.4%) were new and the rest were follow up at our institution. For ascertaining clinical manifestations at presentation, their paper medical records were reviewed and patients were interviewed as well for all possible clinical manifestations. The mean age of patients was 26.2 ± 7.9 years. Disease duration was ascertained by onset of constellation of symptoms suggestive of SLE. The mean time to diagnosis from onset of disease related symptoms was 3.42 ± 1.34 years. Positive family history of autoimmune disorders were elicited in 7 patients.

**Fig.1:**
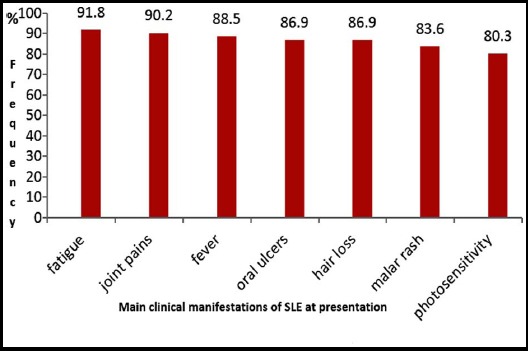
Frequencies (in percentages) of clinical manifestations at presentation in patients with SLE.

Fig.1 shows the frequency of symptoms at the time of presentation. Fatigue was the most common presenting feature in 56 (91.8%) patients, followed by joint pains in 55 (90.2%), fever in 54 (88.5%), oral ulcers and hair loss in 53 (86.9%). Malar rash was present in 51 (83.6%) and photosensitivity in 49 (80.3%).

**Fig.2:**
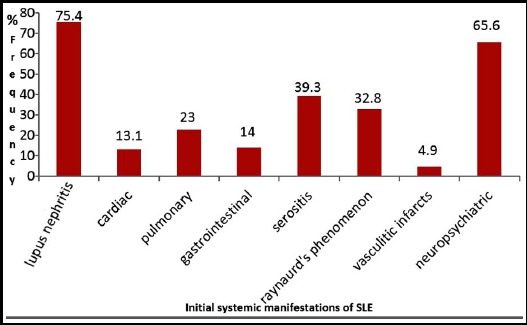
Frequencies (in percentages) of systemic manifestations of SLE patients at presentation.

Other manifestations at initial presentation included lupus nephritis in 46 (75.4%), serositis in 24 (39.3%), Raynaurd’s phenomenon in 20 (32.8%) and vasculitic infarcts in 5 (8.2%) patients. Reported frequencies of neuropsychiatric, cardiopulmonary and gastrointestinal manifestations were in 40 (65.6 %), 20 (32.8%) and 14 (23%) respectively (Fig.2). Ten (16.4) patients presented with ocular symptoms, two patients (3.3%) presented with mononeuritis multiplex, one (1.6%) with acute pancreatitis and pulmonary haemorrhage.

**Fig.3:**
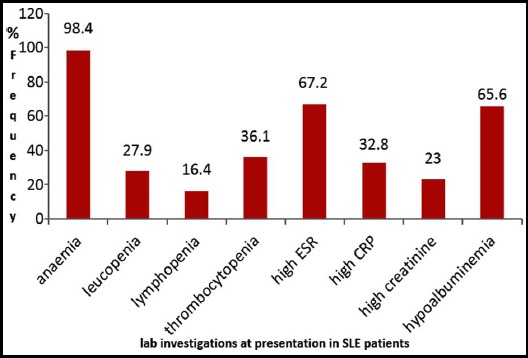
Frequencies (in percentages) of laboratory investigations at presentation in patients with SLE.

Among labatory investigations, anemia was the most frequent finding in 60 (98.4%) patients (Fig.3). Among autoimmune workup in this study population, ANA was positive almost universally in 59 (96.8%) patients. About half of our patients had antibodies to SM nuclear antigen, which is higher than most of the available data of Pakistan. This may reflect a different genetic background of our patients compared to the others.

Comparison of presenting clinical manifestations was made between our study population and other studies carried out in Northern parts of country (Islamabad) and in central Punjab (Pakistan) as shown in Table-I.

**Table-I:** Comparison of presenting clinical manifestations found in this study with that found in studies carried out in central Punjab and Northern part of Pakistan (Islamabad).

*Clinical manifestation*	*Present study n=61 (%)*	*Central Punjab (Pakistan) study n=65 (%)*	*p-value*	*Northern Pakistan (Islamabad) study n=50 (%)*	*p-value*
Fever	54 (88.5)	39 (60.0)	<0.0002	50 (100)	0.01
Fatigue	56 (91.8)	NA	-	50 (100)	0.05
Malar rash	26 (40)	51 (83.6)	<0.001	32 (64)	0.02
Discoid rash	10 (16.4)	13 (20)	0.60	6 (12)	0.51
Alopecia	53 (86.9)	39 (60.0)	0.0001	17 (34)	<0.0001
Arthritis	55 (90.2)	23 (35.38)	<0.001	49 (98)	0.09
Photosensitivity	49 (80.3)	15 (23.0)	<0.001	18 (36)	<0.001
Serositis	24 (39.3)	23 (35.38)	0.64	6 (12)	0.001
Pericardial effusion	11 (18.0)	12 (18.46)	1.0	NA	-
Pleural effusion	8 (13.1)	15 (23)	0.15	NA	-
Renal	46 (75.4)	44 (67.69)	0.33	19 (38)	<0.0001
Neurological	40 (65.5)	13 (20.0)	<0.001	7 (14)	<0.001
Pulmonary	14 (23.0)	31 (47.69)	0.003	NA	-
Anemia	60 (98.4)	55 (85)	0.006	11 (22)	<0.0001
Lymphopenia	10 (16.4)	39 (60.0)	<0.001	NA	-
Thrombocytopenia	22 (36.1)	23 (35.38)	0.92	NA	-
ANA	55 (90.0)	57 (88)	0.66	50 (100)	0.024
Anti dsDNA	52 (85.2)	38 (58.0)	0.0001	32 (64)	0.009
Anti Sm	16 (26.2)	30 (46)	0.02	8 (16)	0.19

There was statistically significant differences in fever, fatigue, malar rash, alopecia, oral ulcers, photosensitivity, arthritis and neurological involvement. (p-value < 0.05) between our population and the other two above mentioned study groups. Statistically significant difference was also noted in serositis in comparison with study population of northern part of Pakistan (p-value < 0.05), while it was not so with the previous study from the same region. Renal manifestations showed statistically significant difference only with Islamabad study (p-value <0.001). Frequencies were comparable in all study groups for anaemia and anti dsDNA. Statistically significant difference was present in antiSm (p-value < 0.02) and ANA (p-value < 0.02) in comparison with Islamabad study and with central Punjab study respectively.

## DISCUSSION

This study is unique because it not only shows the pattern of initial clinical manifestations of SLE but also presents a comparison with the previous studies from Central Punjab) and from northern Pakistan (Islamabad). Most of other studies have focussed on clinical manifestations throughout the course of SLE. To date, three studies on initial clinical manifestations have been carried out in Pakistan.^11^-^13^ The pattern of presenting clinical manifestations observed in these studies have some similarities, however, significant differences were also observed, and it might be because of fact that study from same region was conducted in department of medicine, rather than in a dedicated rheumatology setting.

Most of patients in our study population were young. Mean age at presentation in this study was 26 years, which is comparable with study from central Punjab, and another study from Pakistan reported it to be 31 years.^12^,^13^ More than three forth of our patients were females, which is consistent with other previous studies.^11^-^13^ Similar to Islamabad study, female to male ratio was 4:1, which is in contrast with previous study from Central Punjab, in which it was reported as 16:1.^11^,^13^ Literature search also shows female preponderance, it may indicate genetic susceptibility locus on X chromosome.^14^

Positive family history of autoimmune disorders were elicited in 7 patients, of which 5 were of SLE. These patients would have familial autoimmunity.^15^ Almost every patient presented with fatigue, which is found in other studies as well. Fatigue is one of the most common, non specific, initial presentation of SLE, reported in literature.^16^ Joint pains ranged from arthralgia to intermittent polyarthritis. Fever was accompanying feature with the above two symptoms. Among mucocutaneous manifestations, more than 80% patients presented with oral ulcers, alopecia, malar rash and photosensitivity. These presentations along with fatigue, arthropathy and fever, were significantly different statistically from Islamabad study and central Punjab study. This might be because of presentation of disease at different stages in these studies. Higher frequency of arthritis in this study in comparison with other study from same region might be because of differences in ability to pick synovitis by rheumatologists in our setting. Few patients (16.4%) had discoid rash, which is more or less similar with the two above mentioned studies. Renal involvement is common in SLE and is a significant cause of morbidity and mortality. The clinical presentation of lupus nephritis is highly variable, ranging from asymptomatic hematuria and/or proteinuria to frank nephritic syndrome to rapidly progressive glomerulonephritis with loss of renal function.^17^ Lupus nephritis, which was mainly glomerulonephritis was present in 75.4% of our patients, out of which 24.6% had class IV lupus nephritis, and 18.0% had full-blown nephritic syndrome at presentation. These frequencies were high from Islamabad study, where only 38% patients had renal involvement, however it is comparable with the previous study from central Punjab.

A variety of gastrointestinal symptoms has been described in literature.^18^ Abdominal pain was the most frequent presenting symptom in 14 (23%) of SLE. One patient had pancreatitis at presentation, having raised serum amylase and lipase along with abdominal pain. CT scan of abdomen confirmed the diagnosis after ruling out non-SLE causes of pancreatitis. No gastrointestinal manifestations have been reported in Islamabad study and Central Punjab study.

Cardiopulmonary involvement as initial presentation of SLE is not frequently reported. In total, 23% had pulmonary manifestations at presentation. Half of these patients presented with pulmonary hypertension, 13.1% had pleural effusion, 6.6% had lung parenchymal involvement and one patient was found to have pulmonary haemorrhage. A relatively high cumulative rate of pulmonary involvement (61%) has been described in study from central Punjab, but it failed to reach a statistical significance.

In the present study, 13.1% had cardiac involvement, half of these patients had valvular diseases at presentation. The frequency of pericardial effusion (18%) was comparable with the other study of central Punjab, however it was higher than that of Islamabad study. It is pertinent to mention that echocardiography was only done on clinical suspicion of pericardial effusion and thus may not be the true reflection of frequency shown in our study.

Neuropsychiatric SLE consists of a broad range of neurologic and psychiatric manifestations. The frequency of neurological involvement (65.6%) was higher in contrast with other two studies and this was statistically significant difference.

Among atypical clinical features at presentation, four (6.6%) patients presented with unilateral deep vein thrombosis and would have impaired fibrinolysis.^19^ Three (4.9%) patients had vasculitic infarcts and gangrene. Four (6.6%) had generalized petechial rash at presentation, which was secondary to thrombocytopenia.

In this study population, anemia was found to be universal finding (98.4%), it matched with previously recorded data. No patient had aplastic anemia in this study, 27.9% had leucopenia and 36.1% had thrombocytopenia on initial presentation. There was no significant difference in these haematological findings, as compared to other studies.

Positivity of ANA with indirect immunoflourence testing approaches above 95% using HEP-2 cell line.^20^ We found ANA positivity in 90.2% of patients and it is almost similar to previous studies.

### Limitations of the study

A cohort of 61 patients is rather small and some findings with lower incidence can be attributed to chance. There might be recall bias when patients were interviewed about initial symptoms. Being a single tertiary care hospital based study, these results cannot be generalized. The difference of presentation in the same region signifies that SLE has heterogenous presentation and true frequencies of SLE, can only be obtained by conducting a community based study.

## CONCLUSION

In this study, majority of patients presented with combination of fatigue, fever, rash and arthritis. Almost three-fourth of patients had renal manifestations at initial presentation. Delay in identifying such findings can lead to fatal morbidity and mortality. Therefore, it is important for clinicians to have high index of suspicion for SLE, when patients present with above symptoms, as other manifestation of SLE may appear later in course of disease.
